# A Case of Posterior Reversible Encephalopathy Syndrome Associated with Gilenya^®^ (Fingolimod) Treatment for Multiple Sclerosis

**DOI:** 10.3389/fneur.2015.00039

**Published:** 2015-03-04

**Authors:** Hans Lindå, Anders von Heijne

**Affiliations:** ^1^Neurology Unit, Division of Internal Medicine, Karolinska Institute, Danderyd Hospital, Danderyd, Sweden; ^2^Neurology Clinic, Sophiahemmet, Stockholm, Sweden; ^3^Department of Radiology, Danderyd Hospital, Danderyd, Sweden

**Keywords:** MS, posterior reversible encephalopathy syndrome, Gilenya, fingolimod

## Abstract

We describe posterior reversible encephalopathy syndrome (PRES) in a woman with multiple sclerosis treated with Gilenya^®^ (Fingolimod). The first symptoms appeared after 21 months of fingolimod treatment. She experienced headache, altered mental status, cognitive deficits, seizures, and visual disturbances. Not at any time during the course of the disease could any signs of infection or rheumatic disorder be detected. Test for anti-neuronal antibodies was also negative. Her blood pressure was normal. MRI showed widespread cortical and subcortical changes with some mass-effect in the temporo-occipital-parietal lobes in the left hemisphere. Contrast enhancement was seen in the leptomeninges and, in addition, there were no areas with restricted diffusion and no signs of hemorrhage. Her condition deteriorated until fingolimod was discontinued. Slowly her condition improved and after 8 months, the only symptoms that remained were two small, non-corresponding, right inferior scotomas. We believe that all symptoms, the clinical course, and the MRI findings in this case can all be explained by considering PRES, a probably rare, but serious, side effect of fingolimod treatment.

## Case Presentation

In September 2005, a 38-year-old woman suddenly experienced diplopia. Examinations in her homeland revealed MRI lesions typical for multiple sclerosis (MS). Analysis of the cerebrospinal fluid showed oligoclonal IgG bands and a high IgG index, but no pleocytosis. However, no treatment was initiated at that time. She then had her second relapse in July 2006 with numbness in both legs. All these symptoms disappeared in 5 weeks. Betaseron^®^ (Interferon beta-1b) was initiated in January 2007. In July 2007, she had moved to Sweden and was examined for the first time in our hospital. At that time, she herself did not experience any clinical symptoms, but she had signs on the neurological examination. All tendon reflexes were slightly exaggerated on the right side of the body, the sense of vibration was slightly decreased on the right foot and slight gaze-dependent nystagmus was seen. An MRI scan in August 2007 showed multiple lesions typical for MS of both the brain (20 lesions) and spinal cord (10 lesions). She continued her interferon beta-1b treatment. A new MRI scan in June 2008 did not show any new lesions. In January 2009, she experienced numbness in her left arm and MRI showed a new lesion in the thalamus. Due to mildly elevated liver transaminase levels and the new relapse, interferon beta-1b treatment was discontinued and Copaxone^®^ (Glatiramer acetate) was initiated. A new MRI scan in November 2010 displayed five new lesions and one of them showed contrast enhancement, but she had not experienced any new neurological symptoms. Since she was afraid of progressive multifocal leukoencephalopathy (PML), Tysabri^®^ (Natalizumab) was not initiated (see more below). Instead, she continued her glatiramer acetate treatment. A new MRI in May 2011 showed no new lesions. Neurological examination in December 2011 showed lost sense of vibration in both feet and exaggerated reflexes in both legs.

## Gilenya^®^ (Fingolimod) Therapy

**Figure 1:**
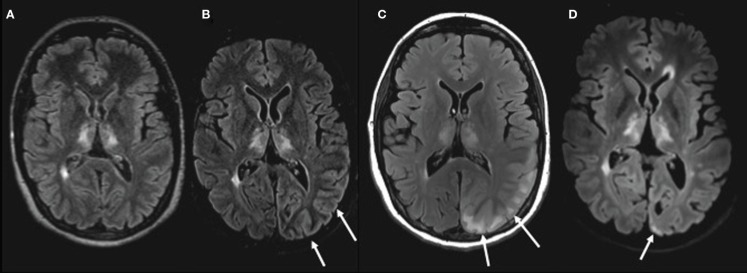
**MRI of the brain performed with FLAIR sequences on four different scanners**. **(A)** Before PRES in July 2013 (3D FLAIR 1,5T). **(B)** First MRI on day 4 during PRES with subtle cortical edema (arrows; 2D FLAIR 1,5T). **(C)** Second MRI on day 24 during PRES with cortical and subcortical vasogenic edema (arrows; 2D FLAIR 3T). **(D)** Follow-up MRI May 2014 with small superficial residual changes (arrow) in part of the cortex (3D FLAIR 3T). Chronic neuroinflammatory lesions are seen in the thalami and periventricular white matter.

**Figure 2:**
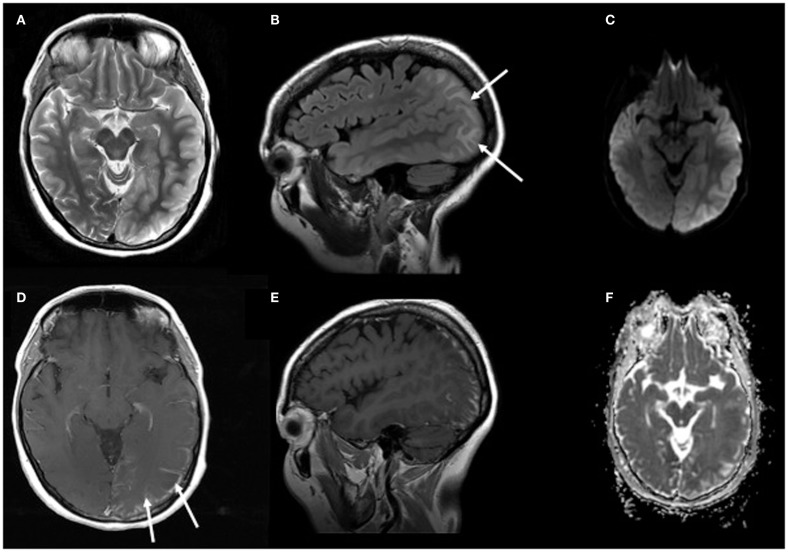
**MRI of the brain on day 24 of PRES**. Images show axial T2 **(A)** and sagittal FLAIR **(B)** with cortical edema and T1 with contrast enhancement **(D,E)** of the leptomeninges. Right column images show DWI **(C)** and ADC **(F)** with vasogenic edema, but without areas with restricted diffusion.

Because of the worsening of symptoms, fingolimod at a dose of 0.5 mg once daily was initiated in December 2011. The first MRI after the initiation of fingolimod was done in January 2012 and showed two new lesions, but no contrast enhancement was seen. An MRI in July 2012 showed no new lesions. In March 2013, the sense of vibration had normalized, but reflexes were still exaggerated in both legs. In July 2013, MRI displayed no new lesions. On September 6 2013, she experienced symptoms of a urinary tract infection, and because of that she contacted her general practitioner. She was then treated with Selexid^®^ (Pivmecillinam) for 3 days and symptoms ameliorated. A family member had communicated with her on September 15 and reported that everything had been completely normal. On September 17, she again came in contact with her general practitioner. She had been found disoriented outside her home. She was at that time fully awake, but she had some mild speech difficulties and apraxia. She was immediately sent in to the nearest hospital, and not to our hospital. At that hospital, some kind of CNS infection was suspected. However, blood samples did not show any abnormalities and she had no fever or any other signs of an ongoing infection. Also, rheumatological markers for inflammation/vasculitis were negative. A first lumbar puncture was not done until September 21, and a second on October 3. No abnormalities were seen in any of the CSF (cerebrospinal fluid) analysis. No pleocytosis, no rise in lactic acid or albumine levels could be seen. Glucose levels were also normal. Interleukin 6 and 10, beta-Amyloid, and Tau levels were also normal. PCR was negative for *Mycobacterium*, Herpes simplex 1 and 2, Varicella zoster, enteroviruses, and JC-virus. No *Borrelia* or syphilis antibodies were present. Fungus screening in the CSF was also negative. In addition, anti-neuronal antibodies were negative in both CSF and blood samples. MRI was, by unknown reasons, not done until September 21 and was at that time described as not changed from an earlier MRI done 2 months before (Figure 1). Her condition had, however, deteriorated. She experienced a headache with mild light sensitivity. She was somnolent and when she was awake she had clear speech difficulties and was disoriented. EEG was abnormal with generalized rhythmic slow activity of delta frequency within the fronto-temporal regions, but no epileptic discharges were seen. However, on September 25, she had a generalized epileptic seizure. Her symptoms then continued to worsen. She suffered from lethargy and had visual hallucinations. Delusions were present and she was still disoriented. At this time and also earlier, no hypertension was at hand, blood pressure had always been around 120/80. Fingolimod had by unknown reasons not been discontinued. A new EEG on September 26 showed generalized slow activity over the whole left hemisphere, and in the frontal region also episodic rhytmic delta activity with triphasic like appearance was seen. Still, no epileptic discharges were present. In spite of the lack of epileptic activity on the EEG, the cause of all symptoms was thought to be primarily epileptic. Anti-epileptic medication was initiated (Keppra^®^, Levetiracetam). On October 2, our hospital was contacted for the first time and fingolimod was then without any delay discontinued since a rare side effect was suspected. At that time, also a CT brain angiography was done and, as expected, no signs of vasculitis were seen. Already a week after discontinuation of fingolimod, the patient had slightly improved. She was more awake and responded to questions, but she had still speech difficulties and was confused. On October 11, a new MRI scan showed widespread cortical and subcortical changes with some mass-effect in the temporo-occipital-parietal lobes in the left hemisphere (Figure 2). Contrast enhancement was also seen in the leptomeninges. There were no areas with restricted diffusion and no signs of hemorrhage. Retrospectively, subtle cortical edema could now be seen in the same areas in the MRI scan from September. In addition, hyperperfusion could also be seen in the same areas. A new EEG was done in October 18, but no changes were seen compared with the registration on October 2. On October 23, the patient was moved to another hospital for rehabilitation. At that time, she had still right homonymous hemianopia. She had also still some speech difficulties and cognitive deficits, but now she was fully oriented and had no visual hallucinations. In November, acute intermittent porphyria was also ruled out as a possible cause of her present condition. She continued to improve and on January 27 2014, she could speak without problems and the visual fields were nearly normal, only a right inferior quadrantanopia remained. A visual examination on April 15 showed only two small, non-corresponding, right inferior scotomas, which do not obstruct her ability to drive a car. She started to work halftime in May, and fulltime in June. An MRI on May 15 showed unchanged neuroinflammatory lesion load and superficial residual cortical lesions in parts of the occipital lobe. When the clinical course and the imaging findings were analyzed at our hospital, we found them highly consistent with posterior reversible encephalopathy syndrome (PRES).

## Background

### Posterior reversible encephalopathy syndrome

Posterior reversible encephalopathy syndrome in general is a relatively rare clinico-radiological entity first described in 1996 (1). Clinically, it is characterized by a subacute onset of headache, altered mental status, seizures, visual disturbances, and occasionally other focal neurological signs (2, 3). Radiologically, there are signs of vasogenic edema bilaterally in the white matter of the parieto-occipital lobes, but changes can also be seen in frontal and temporal lobes, brainstem, cerebellum, and in cortical as well as deep gray matter (3–5). It is most often associated with other medical conditions such as, for example, hypertensive crisis, renal disorders, and eclampsia. However, different medications such as immunosuppressive, chemotherapeutic, and cytotoxic agents are also associated with PRES (2, 3). The underlying pathophysiology is still unknown, but there are many theories. A consistent feature in PRES seems to be dysfunction of the cerebral autoregulation and impaired cerebrovascular blood–brain barrier (6). Cerebral autoregulation is achieved by vasoconstriction or vasodilatation of resistance arterioles, resulting in a constant blood flow to the brain. In the case of significant hypertension, a rapid rise in blood pressure that will overcome the capacity of the normal autoregulation could produce vasodilatation, with a resulting hyperperfusion and/or regional vasoconstriction of the brains arteries. This hyperperfusion could then cause endothelial dysfunction, resulting in an extravasation of proteins and fluid, i.e., causing a vasogenic edema. Arteries in the posterior regions of the brain seem to be especially vulnerable, probably due to a relative lack of sympathetic innervation in these vessels (7). However, there could also be a direct toxic effect on the vascular endothelium followed by a breakdown of the blood–brain barrier and a dysfunction of the vascular autoregulation (3, 6, 8). That could be the case, for example, in eclampsia, sepsis, and in PRES associated with certain medications, especially immunomodulating treatments. Probably, a damaged endothelium could then *per se* contribute to a high blood pressure. Thus, high blood pressure should not be mandatory in PRES and there are indeed normotensive cases (9–11). The fact that a high percentage of patients with PRES have a history of autoimmune disorders supports the idea that affected endothelium plays a role in the pathophysiology of PRES (3, 12). The differential diagnosis of PRES includes various acute neurological conditions such as stroke, cerebral venous thrombosis, encephalitis, and demyelinating disorders (4, 6). PRES is best treated by a prompt reduction of blood pressure, anti-epileptic therapy, and/or withdrawal of the causing agent (4, 6).

### Gilenya^®^ (fingolimod)

On September 22 2010, fingolimod became the first oral disease-modifying drug approved by the US Food and Drug Administration (FDA) to significantly reduce relapses and delay disability progression in patients with relapsing forms of MS. MS patients taking 0.5 mg fingolimod have 54% lower risk of relapses than those taking placebo over 2 years (13). Another study showed that MS patients taking fingolimod had a 52% lower risk of having a relapse than patients taking Avonex^®^ (Interferon beta-1a) over 1 year (14). On March 17 2011, the European Medicines Agency (EMA) approved the drug for use in the European Union. As of February 2014, 91,500 patients have been treated with fingolimod. The most serious adverse effects are cardiac arrhythmias and macular edema. Patients with cardio-vascular risk factors are therefore mostly excluded and a follow-up ophthalmological examination is needed. PRES is probably a rare adverse effect of fingolimod. However, already in a premarketing study (15) one case was reported, but the dose in that case (5 mg) was higher than recommended for use in MS (0,5 mg).

## Discussion

Here, we report, what is to our knowledge, the first occurrence of PRES in an MS patient treated with the recommended daily dose of 0.5 mg fingolimod. The number of fingolimod treated patients with PRES is unknown. However, in a 6 months study comparing fingolimod doses of 1.25 or 5 mg with placebo, one case of PRES was described in the 5 mg group already after 10 weeks of treatment when headache, cortical blindness, ophthalmoplegia, dysarthria, and ataxia developed (15). In contrast to most PRES cases, this patient had no hypertension or renal disorder. MRI features began to improve already 72 h after the discontinuation of fingolimod. However, a residual right homonymous hemianopia corresponding to a left occipital hyperintensity and a mild ataxia remained and did not change after 15 months follow-up (15). Thus, in parallel with that case of fingolimod associated PRES, our patient also had clinical symptoms typical for PRES with visual disturbance, disorientation, and epileptic seizures, but without any hypertension or renal failure. In fact, PRES with no associated hypertension has been described previously (9–11). In our case, it could be speculated that fingolimod had a direct toxic effect on the endothelium. A direct effect of fingolimod on the endothelium has indeed been described. Fingolimod can cause an arterial vasodilation and stimulate NO production (16). Fingolimod has also been shown to modulate the integrity of the endothelium, especially influencing the vascular permeability (17, 18). Moreover, fingolimod could also influence endothelial healing after mechanic injury and impaired angiogenesis (19). The possibly strong effect of fingolimod on arteries was further emphasized in an MS patient with critical vasospasm of the left arm within 7 days after having started treatment with fingolimod (20). In parallel with fingolimod, PRES has also been described in one MS patient treated with natalizumab (21). Natalizumab is a monoclonal antibody and a highly effective treatment for MS. Natalizumab reduces the mean annualized relapse rate over 2 years by 68% compared to placebo and 55% compared to interferon beta-1a (22, 23). As of June 2014, 129,100 patients have been treated with natalizumab. The natalizumab treated patient with PRES had, however, concomitantly an urosepsis and also a very high systolic blood pressure at presentation. Both these conditions can *per se* cause PRES, thus there are other possible reasons in that case (21). In our case, the patient only had a mild urethritis with no increase in white blood cell count and CRP. However, a urinary tract infection could not be completely excluded as a contributing factor for PRES in our case. The clinical presentation of PRES is often atypical, which makes MRI crucial for the diagnosis. In our case, signs of cortical edema could be seen already in the first MRI done 4 days after the initial symptoms but, unfortunately, it was not recognized. This fact emphasizes the importance of an early diagnosis with MRI. The MRI picture in our case was in many ways typical for PRES, with the exception of an asymmetrical presentation. A unilateral MRI appearance has, however, been described before and is estimated to occur in <5% of patients (24, 25). Also, in the PRES case described after 5 mg fingolimod, an asymmetrical MRI appearance was at hand (15).

## Concluding Remarks

Obviously, the new era of highly effective therapies for inflammatory and autoimmune diseases carries risks of probably rare, but serious, side effects. If diagnosis and treatment are delayed, the result could be permanent damage to affected areas of the brain. In parallel with natalizumab, serious side effects emerging in the postmarketing phase have been shown for fingolimod. PML has been shown to be associated with natalizumab (26, 27). PML is a serious demyelinating disease of the central nervous system caused by the human JC polyomavirus (28). As of September 2014, the number of PML cases associated with natalizumab is 492 (Biogen Idec, data on file). Accordingly, it is very important to know about PRES as a possible side effect of fingolimod in order to quickly recognize the condition and discontinue the medication. It is also important to know about the prevalence, but according to Novartis the only information accessible is that the number of PRES cases reported in the postmarketing phase has not increased over time. However, the exact number of PRES cases reported to Novartis is kept confidential. Both PRES and PML (29) are very important to discover in an early phase to minimize residual handicap.

## Conflict of Interest Statement

The authors declare that the research was conducted in the absence of any commercial or financial relationships that could be construed as a potential conflict of interest.
